# A Randomized Controlled Trial on the Safety and Cognitive Benefits of a Novel Functional Drink from a Purple Waxy Corn Byproduct in Peri- and Postmenopausal Women

**DOI:** 10.3390/antiox14101262

**Published:** 2025-10-20

**Authors:** Jintanaporn Wattanathorn, Woranan Kirisattayakul, Woraluk Somboonporn

**Affiliations:** 1 Department of Physiology, Faculty of Medicine, Khon Kaen University, Khon Kaen 40002, Thailand; 2 Research Institute for High Human Performance and Health Promotion, Khon Kaen University, Khon Kaen 40002, Thailand; 3 Department of Radiology, Faculty of Medicine, Khon Kaen University, Khon Kaen 40002, Thailand; woraki@kku.ac.th; 4 Department of Obstetrics and Gynecology, Faculty of Medicine, Khon Kaen University, Khon Kaen 40002, Thailand; wsomboonporn@yahoo.com

**Keywords:** purple waxy corn cob, anthocyanins, cognition, working memory, menopausal women

## Abstract

Fulfilling the demand for functional food with cost safety and environmental sustainability, our novel anthocyanin-enriched functional drink containing the purple waxy corn cob-derived functional ingredient “MP1” showed cognitive enhancing effects with safety in bilaterally ovariectomized rats, a validated model of menopause. Since no clinical evidence that confirms the mentioned effect was available until now, we conducted a two-arm, randomized, double-blind, placebo-controlled, crossover study to confirm the benefits mentioned above. A total of 32 menopausal participants were divided into placebo and MP1 (400 mg) groups, and were subject to a 2-month study period. Safety parameters, working memory and brain components, especially N100 and P300, the negative and positive potentials derived from the event-related potential (ERP) which indicated attention and cognitive processing, together with oxidative stress markers acetylcholinesterase (AChE) and monoamine oxidase (MAO), were assessed at baseline and every month. No serious side effects or toxicity signs were observed. Subjects who consumed MP1 also had decreased N100 and P300 latency, improved working memory and decreased oxidative stress status. Therefore, a byproduct of purple corn can successfully serve as a novel functional ingredient for developing a cognitive enhancer drink with the qualities of safety, cost reduction, and environmental sustainability promotion.

## 1. Introduction

Currently, human life expectancy is increasing due to advancements in technology. The global population of peri- and postmenopausal women has been reported to be around 1 billion [1]. It has been reported that one of the common complaints of women at this age appears to be cognitive problems, particularly attention and memory decline [2,3,4]. Data obtained from working memory tests reveal that menopausal women tend to produce lower test scores and show longer P300 latency than premenopausal women [5,6]. Some studies reported that this condition can be improved by hormone replacement therapy [7,8]. However, the effect of hormone replacement therapy is complex, with inconsistent positive results. A systematic review and meta-analysis study has revealed that there is no evidence of a benefit of any type of estrogen on overall cognitive functioning in older postmenopausal women when given either as short-term or longer-term (up to five years) therapy. In addition, no evidence of a beneficial effect of combined estrogen and progestogen therapy is available [8]. Moreover, estrogen therapy also increases the risk of breast cancer [9], adenocarcinoma [10], and endometrial and ovarian cancer [11,12,13]. Therefore, a novel strategy for attenuating cognitive impairment in menopausal women is required.

Accumulative lines of evidence have demonstrated that the cognitive and memory declines in peri- and postmenopausal women are associated with the erratic fluctuation of female sex hormones such as estrogen levels [2]. The reduction in estrogen, which possesses antioxidant [2] and anti-inflammatory effects [14], leads to an increase in oxidative stress [15] and inflammation [16]. In addition, estrogen deficit also disrupts the dynamic balance of neurotransmitters, particularly acetylcholine (ACh) [17] and monoamine transmitters [18], which in turn induce memory deficits. However, most of the current available products on the market still focus on symptomatic treatment, and fewer products pay attention to the pathophysiology approach. Since the cognitive decline in peri- and postmenopausal women appears to be complex, involving multiple pathways, the pathophysiological approach, which focuses on multi-target strategies, may enhance the efficacy of the intervention [19]. Therefore, searching for a functional ingredient which can act on these multiple targets has gained much attention.

Recent studies have demonstrated that anthocyanins, water-soluble flavonoids, possess antioxidant [20] and anti-inflammation [21] effects. In addition, they can suppress acetylcholinesterase (AChE) and monoamine oxidase (MAO), giving rise to an increase in cholinergic and monoaminergic systems, leading to improvement in cognition and working memory [22]. Although anthocyanins, which serve as a functional ingredient, can increase the market opportunity for products fortified with them, the competition is very high. Therefore, the concept of changing the byproduct to an asset and creating economic opportunities, such as by converting a byproduct that is rich in anthocyanins to a functional ingredient, has gained much attention. This process not only produces a valuable resource such as a functional ingredient but also reduces the unit cost of production, waste products and the emission of greenhouse gases [23]. Based on this concept, and increasing health consciousness of consumers, we have developed an anthocyanin-enriched functional ingredient from purple waxy corn cob, and an innovative drink fortified with the novel functional ingredient, or “MP1” [24].

“MP1” is the first anthocyanin-enriched innovative drink from a byproduct under the biocircular concept. According to our development, green extract was used, and with the application of byproduct recycling, this can reduce the emission of greenhouse gases. Therefore, our product is eco-friendly. It has a neuroprotective effect against neurodegeneration, and enhances memory by suppressing AChE in a rat model of menopause induced by bilateral ovariectomy [23]. We also determined the LD_50_ content and subchronic toxicity of “MP1”, and the results showed that the LD_50_ content of “MP1” was more than 2000 mg/kg BW and the no-observed-adverse-effect level or NOAEL was more than 500 mg/kg BW [24]. Owing to its potential to provide both health benefits and planet benefits, it is a smart functional ingredient. Due to the scientific evidence regarding the health benefits of “MP1” in the rat model of menopause mentioned earlier, we hypothesized that “MP1”, the functional beverage containing a purple waxy corn-derived functional ingredient, should be safe for consumption and enhance cognitive function in peri- and postmenopausal women. To the best of our knowledge, no scientific evidence has been available until now, and clinical evidence is also essential to confirm the safety and health benefits of the novel food, so the randomized, double-blind, placebo-controlled parallel study is essential because it is a gold standard for testing new interventions. Thus, we aimed to conduct a randomized, double-blind, placebo-controlled parallel study to investigate the safety and efficacy of ”MP1”, a novel drink containing a novel anthocyanin-enriched functional ingredient from byproducts of purple waxy corn cob, in terms of its effects on cognition, and its possible underlying mechanism.

## 2. Materials and Methods

### 2.1. Preaparation and Characterization of “MP1”

#### 2.1.1. Preparation of MP1

Cobs of purple waxy corn or *Zea mays* (open pollinated) were harvested from the Faculty of Agriculture, Khon Kaen University, Khon Kaen, Thailand, and kindly provided by Asist. Prof. Dr. Bhalang Suriharn, Department of Plant Science and Agricultural Resources, Faculty of Agriculture, Khon Kaen University, Khon Kaen, Thailand. Leaves of the pandanus palm or *Pandanus amaryllifolius* Roxb. were obtained from Amphoe Muaeng, Khon Kaen province, Thailand. Both material resources were cleaned, cut into small pieces and dried by using a universal oven model, UN110 (Memmert Gmbc, Schwabach, Bavaria, Germany), at 60 °C overnight. Then, they were twice extracted with distilled water using the decoction technique. Both extracts were kept at −20 °C in a freezer until use.

**Figure 1:**
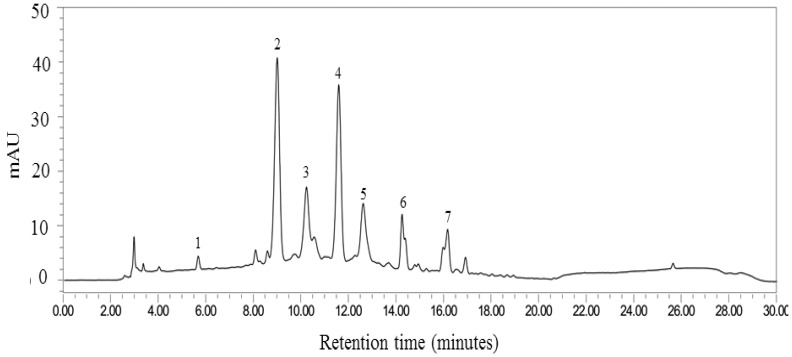
Fingerprint chromatogram of “MP1”. The labelled peaks are gallic acid (peak 1), cyanidin-3-glucoside (peak 2), pelargonidin-3-glucoside (peak 3), cyanidin 3-O-(6”-malonyl-glucoside) (peak 4), cyanidin-3-o-β-glucopyranoside (peak 5), ferulic acid (peak 6) and rutin (peak 7).

To prepare the novel anthocyanin-enriched beverage or “MP1”, a mixture of both extracts at a ratio which provided the optimum benefit (petty patent 10915, registration in 2015 in the Thai language) was used as the functional ingredient. This ingredient was standardized by controlling the content of possible active ingredients, consisting of anthocyanins (cyanidin-3-glucoside, cyanidin 3-O-(6”-malonyl-glucoside), cyaniding-3-o-β-glucopyranoside, pelargonidin-3-glucoside), ferulic acid, rutin and gallic acid, respectively. The concentrations of the substances mentioned above were 3.24 ± 0.01, 2.99 ± 0.02, 2.33 ± 0.01, 2.54 ± 0.01, 0.34 ± 0.03, 0.34 ± 0.01 and 0.18 ± 0.01 mg/mL. The total content of phenolic compounds in the functional ingredient was around 129.25 ± 2.52 mg/L gallic acid equivalent (GAL)/mg. The fingerprint chromatogram is shown in Figure 1. The functional ingredient was prepared as MP1 with a method mentioned earlier elsewhere [24,25]. Briefly, the combined extract (0.25% *w*/*v*), sucralose (0.75% *w*/*v*), lime (1% *v*/*v*), salt (0.025% *w*/*v*) and water (97.975% *v*/*v*) were mixed and boiled until the temperature reached 100 °C, then maintained at that temperature for 15 min. Following this step, 160 mL of the mixture was poured into an aseptic opaque glass bottle which was sterilized by being subjected to a 15 min boiling process. After cooling down, all bottles were sealed and stored at 4 °C. Both the placebo and MP1 contained similar ingredients except for the combined extract of purple waxy corn cob and leaf extract from the pandanus palm. To mimic the color of anthocyanins and the fragrance of pandanus palm in the combined extract mentioned earlier, synthetic food coloring and artificial food coloring were subjected to a similar process. To prepare an identical placebo, all ingredients except the combined extract were used, and purple food coloring (Winners, Great Hill Co., Ltd., Chom Thong, Bangkok, Thailand), and pandan fragrance (Winners’s pandan fragrance, Great Hill Co., Ltd., Chom Thong, Bangkok 10150, Thailand) were added to the placebo to mimic the color and odor of MP1.

#### 2.1.2. Characterization of MP1

Each serving of MP1 contained 400 mg of the combined extract of purple waxy corn (*Z. mays*) cob and leaf extract of pandanus palm (*Pandanus amaryllifolius*). The anthocyanin profile of “MP1” was determined by using the high-performance liquid chromatography (HPLC) method. The system consisted of a 515 HPLC pump equipped with a 2998 photodiode array detector (Waters Corporation, Milford, MA, USA). The separation was achieved on a Purospher^®^ STAR RPC-18 endcapped HPLC column (250 × 4 mm; particle size, 5 µm) and a Merck Chromolith^®^ HPLC guard cartridge guard column (Lot No. HX255346) (Merk KGaA, Darmstadt, Hessen, Germany). The mobile phase consisted of methanol (A) and 7.5% acetic acid in deionized (DI) water (B). The gradient elution was carried out at a flow rate of 1.0 ml/min with the following gradient: 0 min, 10% A; 17 min, 70% A; 18–22 min, 100% A; 25, 50% A; 26–30 min, 10%A. Prior to an injection, the sample was filtered by a filter membrane with a pore size of around 0.2 μm (Whatman, Dusseldorf, Germany) and a direct injection of 20 μL to a column. The chromatograms were recorded at 280 nm using a UV detector, and data analysis was performed using Empower software 3 (Waters Corporation, Milford, MA, USA). The fingerprint chromatogram of “MP1” assessed at the beginning of the clinical trial is shown in Figure 1.

Anthocyanin concentrations in “MP1” and the placebo were 25.66 ± 0.32 (total anthocyanins 4.11 mg/serving) and 0.33 ± 0.00 mg/L of cyanidin-3- glucoside equivalent (CGE). The energy content per serving of MP1 was 3.85 kcal. The contaminations with both microbials and heavy metals were within the allowable limits. Beyond anthocyanins, we also rechecked the concentration the biological activities related to the pathophysiology of cognitive decline, such as antioxidant activity, before commencing the clinical trial study, and the results are shown in Table 1. The IC50 results for the DPPH assay of MP1 were 56.37 ± 0.45 and >2500 μg/mL, respectively, whereas the FRAP assay showed the values at 602.40 ± 2.33 and 5.49 ± 3.33 μM L-ascorbic acid equivalent.

To assure the stability of “MP1” throughout an 8-week study period, we determined the chemical marker contents, such as total phenolic compounds and anthocyanins, which were used for controlling the standard quality of “MP1”, and the biological activities related to the pathophysiology of cognition, such as antioxidant activity [24]. Since the prolonged storage time could lead to a decline in the intensity and prevalence of desirable flavors [25], and the Brix together with the acidity of the product exerted a great influence on consumer acceptance [26], we also determined the changes in the Brix and acidity of ”MP1” throughout the study period. Based on the information mentioned earlier, the stability under 4 °C without light was determined throughout a 3-month study period which covered our clinical trial period (2 months). The results are shown in Table 2.

The current data confirmed that anthocyanin content in “MP1” within the 2-month study period was around 320 mg (the reduction was around 80 mg within 2 months), which still remained within possible therapeutic levels (the effective range according to our previous data showed that the human equivalent dose should be in the range of 194.4–389.4 mg.) Moreover, the Brix and acidity ratios, which played an important role in flavor acceptability, also showed less of a change, so during the study period they appeared to have less of a confounding effect on the changes in quality of the product.

#### 2.1.3. Dose Calculation for Human Application

The dose that was planned for use in clinical trial studies, or the human equivalence dose (HED), was calculated based on our previous preclinical data [6,24,27] according to the following equation [28].

HED = Animal dose (mg/kg) × [Animal Km/Human Km]

Animal effective dose = 40 mg/kg; Animal Km = 6; Human Km = 37

Therefore, the HED was 6.49 mg/kg/day or around 6.5 mg/kg BW. Based on the average weight of the target population group being around 60 kg, the concentration of “MP1” that should be applied per day was 390 mg or around 400 mg. According to this study, the amount of “MP1” used was 160 mL/serving, and it contained 400 mg of the functional ingredient.

### 2.2. Clinical Trial Study

This study was a single-center, randomized, placebo-controlled, double-blind, crossover trial in healthy perimenopausal and postmenopausal women. The study was registered in the ClinicalTrials.gov Protocol Registration and Results System (PRS) (NCT02567760). The study was carried out in accordance with the ethical principles enshrined in the Declaration of Helsinki (1996) and Good Clinical Practice (GCP) guidelines. The study protocol was approved by the Committee of the Center for Ethics in Human Research (CEHR), Khon Kaen University (HE571329; approval date was 29 December 2015). All subjects provided written informed consent before participating in this study, and the blank written consent form is provided in Supplementary Material File S3.

#### 2.2.1. Subjects

Peri- and postmenopausal women were recruited from the Menopause Clinic, Department of Obstetrics and Gynecology Department, Faculty of Medicine, Khon Kaen University. Eligibility criteria included healthy women at the age of between 45 and 60 years old who were classified as peri- or postmenopausal women in the Northeast of Thailand. In the case of natural menopause, the menstrual cessation bust be less than or equal to 5 years before the study. Exclusion criteria were participants with the following criteria:

(1) Subject to cardiovascular disease, respiratory disease, neuropsychological diseases, head injury, diabetes, cancer, autoimmune disease, liver disease or hematologic disorders;
(2) Treated with hormonal replacement;
(3) Taking psychoactive drugs or nutraceutical compounds influencing the function of the central nervous system;
(4) With a previous hysterectomy and/or oophorectomy;
(5) Alcohol user or current smoker.

Participants could withdraw from the trial at any time. In addition, the failure of subjects to adhere to the protocol requirements and the presence of side effects or adverse effects and toxicity signs were also included in the withdrawal criteria.

#### 2.2.2. Sample Size Calculation

The sample size used in this study was calculated based on the primary outcome of cognitive function derived from the event-related potential (ERP) derived from our previous clinical study in this population group [6]. The calculation was performed based on a 95% confidence interval and a power of 90%; the standard deviation for the control group was 0.9 and that for the treatment group was 1.16. Therefore, the required sample size was 29/arm. However, we expected to have 10% withdrawal, and this gave rise to a sample size around 32/arm.

#### 2.2.3. Randomization and Blinding

Eligible participants were randomly assigned to the placebo or control group and the MP1-treated group by a staff member at the office of the Research Institute of High Human Performance and Health Promotion, who was not involved in the trial assessments, by using block design (block of 4) via a computer. The running code was kept in a sealed envelope by the principal investigator and was to be opened after the data were analyzed or when a medical emergency occurred. Both participants and assessors were blinded until the data were analyzed. Both randomization concealment and blinding were successfully maintained during the study.

#### 2.2.4. Study Design

**Figure 2:**
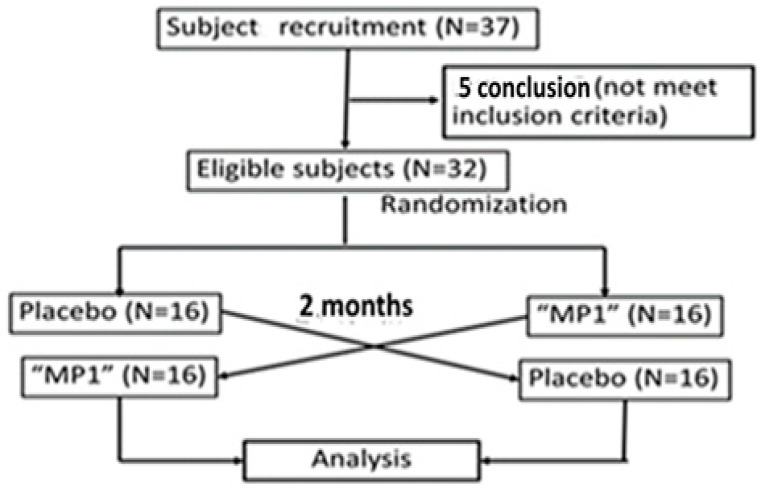
Flow chart of the study.

This study was designed as a one-center, randomized, double-blind, placebo-controlled crossover study and 2  ×  2 trial with an allocation ratio of 1:1, as shown in Figure 2. The subjects were recruited at the Menopause Clinic, Faculty of Medicine, Khon Kaen University, by the staff of the clinic and Ms. Kirisattayakul, W. After being given an interview and a physical examination by the physician for the project, and providing a written consent form, the subjects were randomly allocated to either the placebo or the MP1-treated group (N = 16/arm). All subjects were requested to consume 160 mL of the assigned substances (both the placebo and the functional drink provided a total energy level of 3.85 kilocalories per serving) within 10 min once daily 30 min before breakfast for 2 months. The primary outcome of the study was event-related potential (ERP). The parameters included the alterations in both latency and amplitude of P300 and N100. The secondary outcomes consisted of working memory, serum oxidative stress, serum total phenolic compound level and the activities of both acetylcholinesterase (AChE) and monoamine oxidase (MAO). The consumption safety was also monitored by using hematological changes and blood chemistry profiles as parameters. All parameters were measured at baseline or prior to the intervention, and every month until the end of the 2-month study period. After a 2-week washout period (the half-life of anthocyanins is around 132.6 min [28], so 2 weeks were enough to eliminate the carrying effect), the second session was initiated and the placebo-treated group had to consume “MP1”, whereas the “MP1”-treated group had to consume the placebo. At 1 month after the cessation of the second session, assessments of ERP and working memory were also performed. During the study period, all participants were requested not to consume other functional foods or beverages, and maintained their normal lifestyles. Compliance was monitored by counting the bottles of the functional drink between each visit. To decrease confounding errors, physical activity and food consumption were monitored by using the questionnaires provided in the Supplementary Files S1 and S2.

### 2.3. Assessment of Cognitive Function

The cognitive function domains, especially attention and cognitive processing, were assessed by using event-related potential, as mentioned in detail elsewhere [6,29,30]. In brief, the amplitude and latency of the N100 and P300 brain wave components derived from ERP during the response to the auditory oddball paradigm method were measured. Each subject was provided with sequences of sounds consisting of high-frequency and low-frequency sounds. During participation in the experiment, the subject was requested to differentiate the two different sounds (650 Hz and 1 KHz) mentioned earlier, and pressed different buttons as instructed. The sounds were generated with an interstimulus interval of 1250 msec and an intensity of 60 dB, approximately 85 and 15% of the total stimuli, and detected through headphones (NordicNeuroLab, Milwaukee, WI, USA). During this process, selective attention and cognitive processing were required, and activities that indicated the mentioned parameters were monitored by a 10–20 system of electroencephalography (Neuroscan, Inc., Sterling, Herndon, VA, USA).

### 2.4. Working Memory Assessment

In this study, we assessed 4 domains of working memory consisting of power of attention, continuity of attention, quality of memory and speed of memory by using a computerized battery test [6,29,30]. The battery test comprised word presentation, picture presentation, simple reaction time, digit vigilance, choice reaction time, spatial working memory, numeric working memory, delayed word recognition and delayed picture recognition tests. The percentages of response accuracy and response time in the tests mentioned above were monitored. Response times for the simple reaction time, digit vigilance and choice reaction time tasks were used as parameters indicating power of attention, whereas percentages of response accuracy derived from the digit vigilance and choice reaction time tasks were used as parameters indicating continuity of attention. The quality of memory and speed of memory were derived from the percentages of response accuracy and response times of the following tests: the word recognition, delayed picture recognition, spatial memory and numeric working memory tasks.

#### 2.4.1. Word Presentation

In this test, fifteen words were presented on the VGA color monitor in sequence with an interstimulus interval of around 1 s. Then, the subject had to retrieve the word sequence in the correct order.

#### 2.4.2. Picture Presentation

After being exposed to a series of 20 photographs at a rate of 1 every 3 s, with a stimulus duration of 1 s, the subject had to retrieve information from the presentation.

#### 2.4.3. Simple Reaction Time

A set of 50 instances of the word “yes” was presented on the monitor with an interstimulus interval of 1–3.5 s, and the reaction time that each subject spent to press the “yes” response button was recorded.

#### 2.4.4. Digit Vigilance Test

Each subject had to match the digits that were presented at the right corner and at the center of the VGA monitor simultaneously at a rate of 80/min, and pressed the “yes” button as quickly as possible.

#### 2.4.5. Choice Reaction Time

Each subject had to select and press the corresponding button when the word “no” or the word “yes” was presented on the monitor. Each set consisted of 50 trials presented with an interstimulus interval of 1–3.5 s.

#### 2.4.6. Spatial Working Memory

A picture of a house containing nine windows including four illuminated windows was presented on the screen. Then, each subject was shown a set of 36 similar pictures, and the subject had to match the positions of the illuminated windows with the presented picture. After matching, the subject had to press the “yes” or “no” response button as quickly as possible.

#### 2.4.7. Numeric Working Memory

Each subject was shown five digits presented sequentially on the screen, and had to memorize them. Following this step, the subject had to match the presented digits with a series of 30 probe digits presented and press the “yes” or “no” response button as quickly as possible.

#### 2.4.8. Delayed Word Recognition

The original words plus 15 distracter words were presented one at a time in a randomized order. Then, each subject had to match the presentation with the original words by pressing the corresponding button, “yes” or “no”, as quickly as possible.

#### 2.4.9. Delayed Picture Recognition

In this test, each subject was exposed to the original pictures plus 20 distracter pictures in a randomized order. Then, the subject had to match the presentation with the original pictures by pressing the corresponding button, “yes” or “no”, as quickly as possible.

### 2.5. Biochemical Assay

#### 2.5.1. Blood Sample Preparation and Determination of Protein Concentration

Blood was collected and kept in ethylenediamine tetraacetic acid (EDTA) containing tubes to prevent clotting. Then, it was centrifuged with a speed of 3000 rounds per minute (rpm) at 4 °C for 10 min. The obtained plasma was collected and kept at −20 °C until use. The plasma was diluted (1:10 *v*/*v*) with 0.2 M phosphate-buffered saline (PBS, pH 7.4), and protein concentration was quantified by the application of a standard Protein A280 measurement protocol for the NanoDrop instrument (Thermo Fisher Scientific, Wilmington, DE, USA). Briefly, 2 µL of the diluted plasma sample was placed on the measurement pedestal, and the sampling arm was lowered for measurement. The instrument provided a spectral graph, the absorbance value at 280 nm (A280) and the protein concentration, which was expressed in mg/mL.

#### 2.5.2. The Determination of Malondialdehyde (MDA)

MDA assessment was carried out by using colorimetric assays [31]. In brief, an aliquot of plasma at a volume of 0.6 mL, 0.5 mL of 10% trichloroacetic acid (TCA), 0.5 mL of 5 mM ethylenediaminetetraacetic acid (EDTA) and 0.2 mL of 8.1% of sodium dodecyl sulfate (SDS) were mixed and incubated at room temperature for 10 min. Then, 0.5 mL of 0.6% thiobarbituric acid (TBA) was added to the mixture and boiled at 95 °C in a water bath for 30 min. After cooling, the mixture was centrifuged at 4000 rpm for 10 min and the organic layer was taken and the absorbance was measured at 532 nm. Data were expressed as nmol/mg protein. Tetramethoxypropane (0–20 nmol) was used for preparing the standard calibration curve.

#### 2.5.3. Assessment of Scavenging Enzymes

The main scavenging enzymes, including superoxide dismutase (SOD), catalase (CAT) and glutathione peroxidase (GSH-Px), in the brain homogenate were assessed according to a method previously described elsewhere [25]. To determine the SOD activity, 20 µL of plasma was mixed with a mixture that contained 216 mM potassium phosphate buffer (KH_2_PO_4_), 10.7 mM ethylenediaminetetraacetic acid, 1.1 mM cytochrome C and 0.54 mM xanthine solution, pH 7.4, at a ratio of 25:1:1:50. Then, 20 µL of 0.05 units/mL of xanthine oxidase was added, and incubated for 5 min at room temperature. Absorbance was measured at 490 nm using a microplate reader. SOD activity was expressed as units/mg protein.

CAT activity in plasma was determined by mixing 10 µL of plasma, 50 µL of 30 mM H_2_O_2_, 25 µL of 5 N H_2_SO_4_ and 150 µL of 5 mM KMnO_4_ together. After shaking thoroughly, absorbance was measured at 490 nm. Data were expressed as units/mg protein.

GSH-Px activity was monitored using the colorimetric method. In brief, 20 µL of plasma was added to 10 µL of DTT in 6.77 mM potassium phosphate buffer (pH7), 100 µL of sodium azide in 6.77 mM potassium phosphate buffer (pH7), 10 µL of glutathione solution and 100 µL of H_2_O_2_. The mixture was mixed well and incubated for 10 min at room temperature. Then, 10 µL of DTNB was added and absorbance was measured at 415 nm using a microplate reader. GSH-Px activity was expressed as units/mg protein.

#### 2.5.4. Acetylcholinesterase (AChE) and Monoamine Oxidase (MAO) Activity Assessment

AChE activity in plasma was measured by using the method of Ellman and coworkers [32] with a slight modification. A mixture of 200 μL of 0.1 mM sodium phosphate buffer (pH 8.0), 10 μL of 0.2 M DTNB (5, 5′-dithio-bis-(2-nitrobenzoic acid)) and 20 μL of plasma was mixed and incubated at room temperature for 5 min. The absorbance at 415 nm was recorded via a microplate reader (iMark™ Microplate Absorbance Reader). Then, 10 µL of acetylcholine thiochloride (ACTI) was added and incubated for 3 min. The absorbance of the mixture was measured at 415 nm. The activity of AChE was expressed as mmol/min.g protein.

Monoamine oxidase (MAO) activity was determined according to the method of Holt and coworkers with a slight modification [33]. Briefly, 50 μL of plasma was mixed with a chromogenic solution which consisted of 1 mM vanillic acid, 500 µM 4-aminoantipyrine and 4 U/mL of 1-peroxidase in 0.2 M potassium phosphate buffer, pH 7.6. Then, tyramine was added and used as a substrate. After the mixture was subjected to a 30 min incubation period at 37 °C, absorbance at 490 nm was measured with a microplate reader (iMark™ Microplate Absorbance Reader, Bio-Rad Laboratories, Inc, Hercules, CA, USA). The data were expressed as nmol/h/mg protein.

### 2.6. Total Phenolic Compounds Assessment

The total phenolic compound content was determined by using the Folin–Ciocalteau method [24]. Briefly, 20 µL of plasma was mixed with 1.58 mL of distilled water and 0.1 mL of 50% (*v*/*v*) Folin–Ciocalteu phenol reagent (Sigma-Aldrich, St-Louis, MO, USA). After exposure to an 8 min incubation period, 0.3 mL of 20% sodium carbonate was added and mixed well. The mixture was incubated at room temperature for 2 h and the absorbance was measured at 765 nm with a UV-spectrophotometer (Pharmacia LKB-Biochrom4060, Pharmacia LKB Biochrom Ltd., Cambridge, UK). Gallic acid was used as the standard calibration curve with a concentration range from 50 to 600 mg/L. Data were expressed as mg/L gallic acid equivalent.

### 2.7. Safety Evaluation

Participants were requested to fast overnight, and the blood was collected in the morning. The changes in hematology and blood chemistry were assessed at Laboratory unit, Srinagarind Hospital, Faculty of Medicine, Khon Kaen University. In addition, an adverse effect assessment was performed using the adverse effect record. Participants were requested to record adverse events including breast tenderness, hot flashes, vaginal bleeding, leucorrhea, stomachache, heartburn, extreme weight gain and others. Each item was graded by using a score ranging from 0 to 4 (0—not at all, 1—mild, 2—moderate, 3—rather severe, 4—severe).

### 2.8. Statistical Analysis

Data were presented as mean ± SD and were analyzed with IBM SPSS Statistics V21.0 (IBM acquired SPSS Inc, Armonk, NY, USA). Only data obtained from the participants who adhered to all visits were analyzed in this study. Each variable was determined in the normality distribution by using the Shapiro–Wilk test before the statistical analysis. Inter-group comparison at each time point was analyzed by using one-way analysis of variance (ANOVA) or the Mann–Whitney U test. Data which showed normal distribution were analyzed by using ANOVA and the Tukey test whereas the data which failed to show normal distribution were analyzed by using the Mann–Whitney U test. The adverse effects analysis was carried out by using the Chi-square test. The analysis was performed only on the participants who completed all visits in this study. A *p*-value < 0.05 was regarded as a significant difference.

## 3. Results

### 3.1. Participant and Demographic Data

A total of 37 subjects were recruited to participate in this study. After screening by the physician for the project, it was found that the number of eligible participants was 32. During the first session, two participants in the placebo group withdrew from the study due to personal reasons, and the data of these participants were excluded from the analysis. The current data demonstrated that the participants who were randomly assigned to the placebo and MP1 groups failed to show significant differences in demographic data including age, duration since the cessation of menstruation, body weight, body mass index (BMI), blood pressure, heart rate, education or the distribution of peri- and postmenopausal women, as shown in Table 3.

### 3.2. Effect of MP1 on Cognitive Function Domains

**Figure 3:**
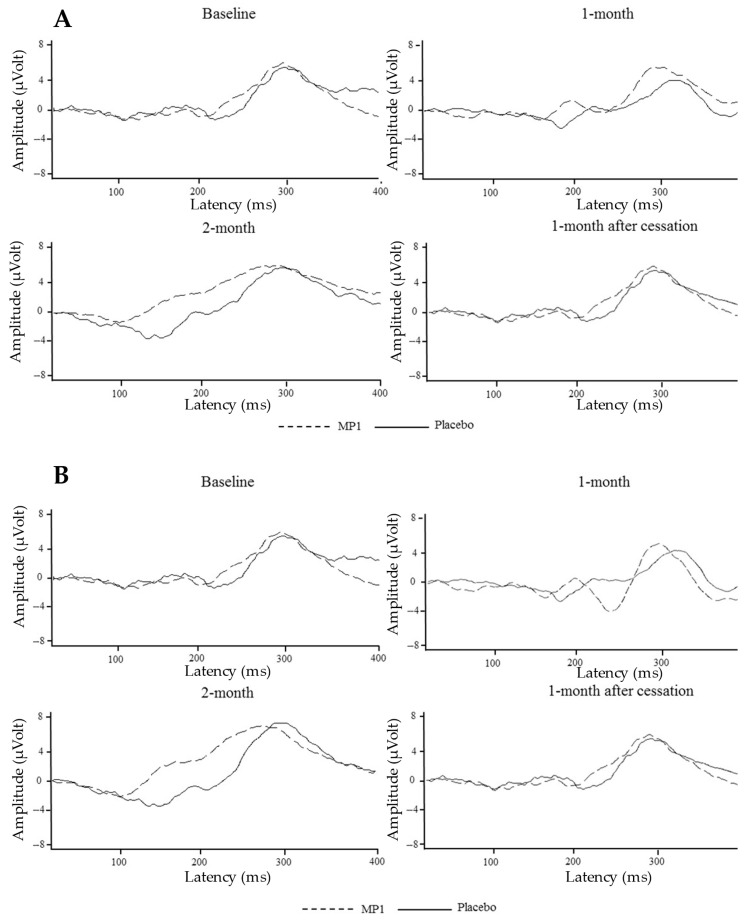
The average auditory ERP waveforms at the C_z_ (**A**) and F_z_ (**B**) electrodes across various treatment periods.

The effects of MP1 on ERP components were assessed after one and two months of intervention, as well as at one month after the cessation of treatment; the results are presented in Table 4 and Figure 3. Interestingly, the “MP1”-treated group exhibited significant reductions in N100 and P300 latencies at the Fz location (*p* < 0.01 and *p* < 0.001, respectively, compared to the placebo group) after one month of treatment. A significant reduction in P300 latency was also observed at the Cz location (*p* < 0.001 compared to the placebo group). When the treatment was extended to 2 months, it was found that the reduction in P300 latency observed at the Fz location was still present (*p* < 0.001 compared to the placebo group). At this period, reductions in latency of both the N100 and P300 brain wave components in the “MP1”-treated group were also observed at the Cz location (*p* < 0.05 and *p* < 0.001, respectively, compared to the placebo group). All changes mentioned earlier were not observed 1 month after treatment cessation, as shown in Table 2 and Figure 3A,B.

The effects of MP1 on working memory at baseline, after 1 month and 2 months treatment and 1 month after cessation were also determined, and the results are shown in Table 5. It was found that after one month of consumption, subjects who consumed “MP1” showed an improvement in % accuracy of choice reaction time (*p*-value < 0.05, compared to the placebo-treated group), and decreased reaction time in the spatial memory task (*p*-value < 0.05, compared to the placebo-treated group). However, these significant changes in the mentioned parameters disappeared when the intervention was prolonged to 2 months and in the follow-up period at 1 month after the cessation of treatment.

### 3.3. Effect of MP1 on Oxidative Stress Status

**Figure 4:**
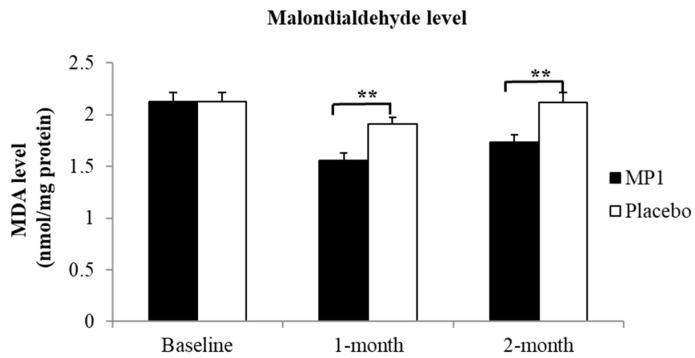
Effects of “MP1” on serum malondialdehyde (MDA) levels at baseline, and after 1 and 2 months of treatment (N = 30/group). Data are presented as mean ± SD. **, *p*-value < 0.01 compared with the placebo group.

**Figure 5:**
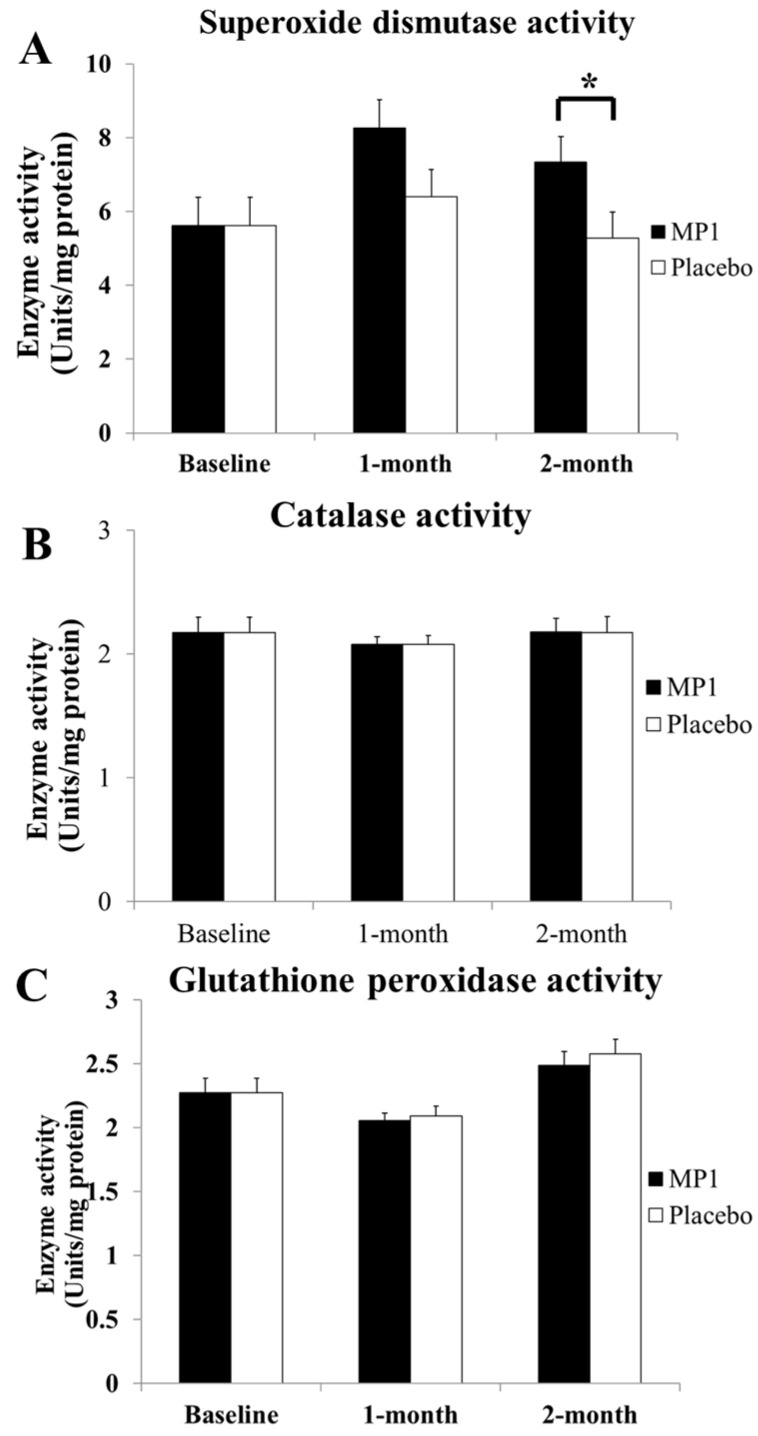
The effects of “MP1” on the activities of the main scavenging enzymes, including superoxide dismutase (SOD) (**A**), catalase (CAT) (**B**) and glutathione peroxidase (GSH-Px) (**C**), in plasma (N = 30/group). Data are presented as mean ± SD. *, *p*-value < 0.05, compared with the placebo group.

Figure 4 demonstrates that subjects who consumed MP1 had decreased plasma MDA levels after one and two months of treatment (*p* < 0.05 for all, compared to the placebo group). The effects of “MP1” on the plasma activities of SOD, CAT and GSH-Px are presented in Figure 5. The results indicated that after two months of intervention, participants receiving MP1 exhibited a significant elevation in SOD activity compared to the placebo group (*p* < 0.05). No other significant effects were observed.

### 3.4. Effect of MP1 on AChE and MAO Activities

**Figure 6:**
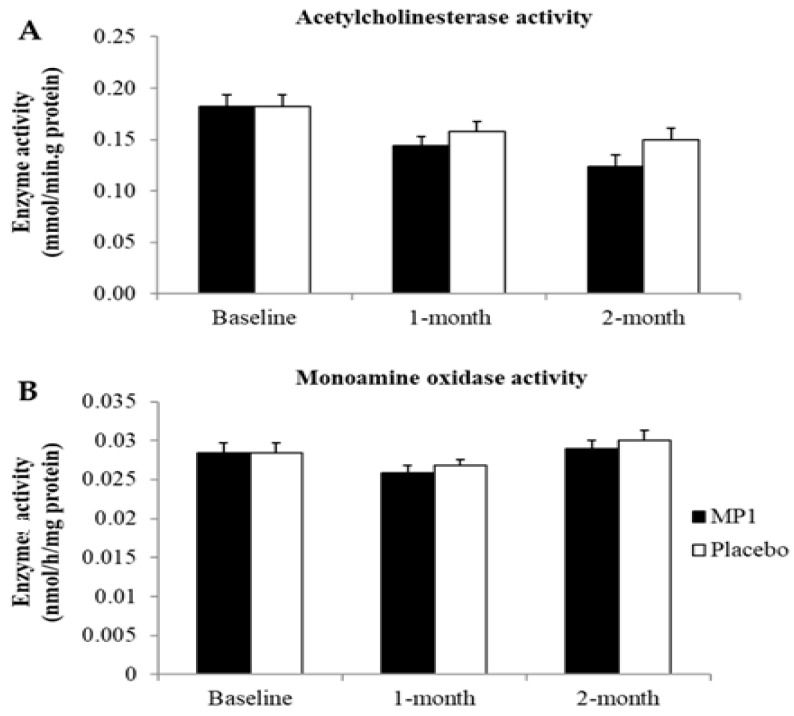
Effect of “MP1” on the activities of acetylcholinesterase (**A**) and monoamine oxidase (**B**) in plasma (N = 30/group). Data are presented as mean ± SD.

**Figure 7:**
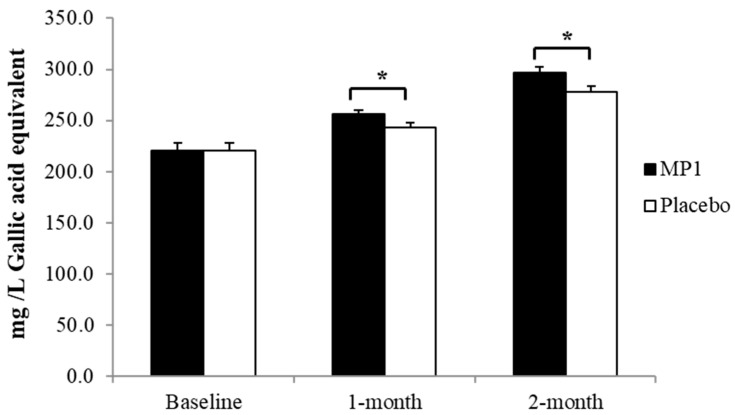
The total phenolic compounds in plasma. *, *p*-value < 0.05 compared with the placebo group (N = 30). Data are presented as mean ± SD. *, *p*-value < 0.05, compared with the placebo group.

Figure 6 and Figure 7 demonstrate the effect of “MP1” on AChE and MAO activities. When compared to the placebo-treated group, no significant changes in AChE and MAO were observed in the MP1-treated group throughout the study period.

### 3.5. Effect of MP1 on Total Phenolic Compounds

The effect of “MP1” on the levels of total phenolic compounds in plasma is presented in Figure 6. A significant elevation in these substances was observed in participants who consumed “MP1” after both one and two months of treatment (*p* < 0.05 for all; compared to the placebo group).

### 3.6. Safety Evaluation

The effect of “MP1” on blood chemistry profiles is presented in Table 6. After one month of consumption, the MP1 group exhibited a significant reduction in bicarbonate levels compared to the placebo group *(p* < 0.01). However, this value still remained in the normal range. No other significant changes were observed, and all values remained in the normal range.

Table 7 shows the effect of “MP1” on hematological changes. No significant differences in hematological parameters were observed between the MP1 and placebo groups after one or two months of consumption. Furthermore, no significant changes from baseline were detected in any parameter throughout the study period.

The adverse events that occurred during the intervention periods are shown in Table 8. The results showed that after consumption for 1 month, eight types of adverse effects were reported in the “MP1”-treated group, including breast tenderness (6.67%), hot flashes (10%), perspiration (6.67%), mood swings (3.33%), dizziness (3.33%), frequent urination (6.67%), extreme weight gain (6.67%) and headache (3.33%), while six types of adverse effects were reported in the placebo-treated group, including breast tenderness (3.33%), hot flashes (26.67%), perspiration (3.33%), dizziness (3.33%), extreme weight gain (3.33%) and headache (3.33%). However, no significant difference between the two groups was observed. In addition, four types of adverse effects were also reported after consumption of “MP1” for 2 months, including breast tenderness (6.67%), hot flashes (3.33%), perspiration (3.33%) and extreme weight gain (3.33%), while three types of adverse effects, including breast tenderness (3.33%), hot flashes (6.67%) and extreme weight gain (3.33%), were also reported in the placebo-treated group. No significant difference between the placebo- and “MP1”-treated groups was present. In addition, no serious effects were observed in the “MP1”-treated group.

## 4. Discussion

The results of the present study clearly reveal that the novel functional drink containing a functional ingredient derived from the mixture of the purple waxy corn cob and pandanus palm leaf extracts improves oxidative stress status by increasing SOD while decreasing the MDA level in serum, as well as reducing N100 and P300 latencies, with no serious toxicity. All hematological and biochemical parameters that changed were found to remain within their respective normal ranges.

Event-related potential, a measurement technique developed based on electroencephalography (EEG), can capture the participants’ EEG activity within a specific time window during performance of the cognitive activity process, thus providing a time-series interpretation of the dynamic changes in the cognitive load [34]. Currently, it has been accepted as an important method of measuring cognitive load in cognitive psychology [35]. P300 latency is recognized as a quantitative, unbiased measure for detecting changes in cognition [36] whereas N100 latency, a short latency generated in the frontal cortex [37], is used for assessing attention [38].The latency of N00 reflects time that is required for the process of stimulus-driven triggering of attention [39], whereas P300 latency indicates the time of a brain’s cognitive response to a significant stimulus such as target information [40]. Our results reveal reductions in both N100 and P300 latencies which in turn suggest that the time requirement for stimuli to trigger attention and the time requirement for cognitive response to target information are reduced. Therefore, MP1 successfully improves the times required for attention and cognitive processing.

We also determine the effect of “MP1” on various domains of working memory. Our results reveal that “MP1” improves response accuracy in choice reaction time, which indicates an improvement in decision-making speed and attention [41]. In addition, “MP1” also improves reaction time in the spatial memory task, which measures speed of recalling and retrieving information concerning the memory of position and location [42]. These results correspond to the improvements in speed of both attention and cognitive processing of target information derived from ERP.

Accumulative evidence demonstrates the negative relationship between cognitive decline and oxidative stress elevation [43,44,45]. Oxidative stress dysregulation characterized by a reduction in SOD can lead to the structural damage of brain cells, which in turn induces neuronal circuit damage, affecting attention and cognitive processing [44,45]. In addition, hypomyelination induced by oxidative stress can also slow cognitive processing speed [46]. Since the main changes in cognition, including attention and working memory, appear to be associated with the speed of cognition, we suggest that “MP1” may enhance SOD, which in turn attenuates an attack of oxidative stress on the lipid component, a principal target of oxidative stress [47,48], leading to the destruction of the lipid components of neurons and myelin sheaths, resulting in improvements in attention and cognitive processing, particularly in the areas of the temporal cortex, especially the hippocampus, and the frontal cortex. Therefore, improvements in the spatial memory task, which is associated with the hippocampus [49], and choice reaction, which is associated with the frontal cortex [50,51], are observed. Our results failed to show significantly different changes in CAT and GSH-Px, which break down hydrogen peroxide (H_2_O_2_) into water (H_2_O) and oxygen (O_2_) [52,53], while they showed a significant increase in SOD, which converts superoxide radicals into hydrogen peroxide (OH) and molecular oxygen (O_2_) [53]. The difference between CAT and GSH-Px is that CAT can act without a cofactor whereas GSH-Px requires a cofactor for its function [51]. Owing to the significant increase in SOD activity without changes in CAT and GSH-Px, it has been suggested that “MP” increases the ability of SOD to covert superoxide radicals into hydrogen peroxide (OH) and molecular oxygen (O_2_). A possible explanation for this phenomenon may be associated with the content of zinc (19.25 mg/kg) in corn cobs [54]. Zinc may act as a cofactor with copper, and may promote proper structure and catalytic function by stabilizing its active site for copper’s catalytic role in the Cu/Zn-SOD enzyme or SOD1 in cytoplasm and other cellular compartments [55]. In addition, anthocyanins may activate the nuclear factor erythroid 2-related factor 2 (Nrf2) pathway to upregulate antioxidant enzymes, especially SOD, because this pathway may upregulate the expression and activity of SOD more than CAT and GSH-Px [56]. Therefore, the SOD activity increases without significant changes in CAT and GSH-Px. Our data are not aligned with some studies which reveal that anthocyanins also increase CAT and GSH-Px [57]. A possible explanation may be the difference in anthocyanin profiles. “MP1” contains cyaniding-3-glucoside (peak 2), pelargonidin-3-glucoside, cyanidin 3-O-(6”-malonyl-glucoside)) and cyaniding-3-o-B-glucopyranoside, while anthocyanins from mulberry contain mainly Cyanidin-3-rutinoside and cyanidin-3-glucoside.

Data obtained from in vitro, preclinical and clinical studies clearly reveal that polyphenols, especially anthocyanins, exhibits antioxidant effects [58,59,60,61], and cognitive enhancing effect [62,63]. Therefore, we suggest that the cognitive enhancing effect of “MP1”, which is rich in anthocyanins, observed in this study may be associated with the contents of polyphenol and anthocyanins in “MP1” because subjects who consumed “MP1” show increases in serum polyphenolic compounds after 1 and 2 months of consumption, and this change shows the tight association with the improvements in cognition and memory. However, a limitation of this study is the lack of plasma anthocyanin data which could confirm the association of cognitive function and anthocyanins, because polyphenolic compounds do not only include anthocyanins.

No toxicity signs or serious adverse effects induced by “MP1” are observed. Taking all the data together, the current results suggest that the beverage containing the functional ingredient derived from the mixture of the extracts of the corn cob, a byproduct of purple waxy corn, and the leaf extract of pandanus palm is safe and successfully improves cognition and working memory. Therefore, the byproduct of purple waxy corn can be utilized as a natural resource for developing an anthocyanin-enriched functional ingredient which is safe for consumption and easy to achieve. The utilization of this byproduct can save costs and decrease waste and pollution of the environment.

Therefore, this study is the first study to demonstrate that the anthocyanin-enriched functional ingredient is safe and can be used for developing the cognitive enhancing supplement “MP1”. This product clearly demonstrates consumption safety and cognitive enhancing effects in peri- and menopausal women. The underlying mechanism may occur partly via the reduction in oxidative stress status.

Although this study shows the potential of the novel functional ingredient and beverage ”MP1”, larger sample sizes of both males and females are required to confirm the positive modulation effect and the extent of application. In addition, an exploration to analyze the differences in dietary fiber, which may also modify the gut–brain axis and give rise to cognitive enhancing effects, should also be conducted. Although we also focus on the amount and types of food consumption in this study, there was no deep exploration of the differences between foods that can exert either pre- or probiotic effects in the food consumed among the groups. This may be an opportunity to exert confounding effects on cognitive function by modulating the gut–brain axis. Thus, further research to control these factors should be required to confirm this positive beneficial effect. Moreover, a larger sample size is required to confirm this potential benefit.

## 5. Conclusions

The current study clearly reveals that functional ingredients from food waste such as the cob of purple waxy corn are safe for utilization as natural resources for developing functional beverages such as “MP1”. “MP1” can improve attention and working memory partly via the reduction in oxidative stress. The utilization of byproducts such as purple waxy corn cob may save costs of production and decrease pollution. Since the extraction process is a clean extraction process, it decreases pollution, and it effectively improves attention, cognitive processing and working memory. Therefore, “MP1” can provide benefits to both human health and the planet, and can be recognized as a “smart food”. However, this study is still in the pilot phase and a proof of concept study, larger sample size and long-term effect study are required before generalization for application. Moreover, the functional ingredient used in this study does not only contain anthocyanins, so the health benefit obtained from other substances and interactions among various ingredients still cannot be omitted. A future in-depth exploration is required to be sure about the possible ingredients required for the health benefits induced by the extracts and “MP1”, and this information can be used for controlling the quality of the product.

## Figures and Tables

**Table 1:** The biological activities of MP1 and the placebo.

Parameter	MP1	Placebo
Total phenolic compound (mg/L GAE)	184 ± 1.91	3.78 ± 2.22
Anthocyanin content (mg/L CGE)	25.66 ± 0.32	0.033 ± 0.00
FRAP activity (μM L-ascorbic acid equivalent)	602.40 ± 2.33	5.49 ± 3.33
DPPH radical activity (IC_50_ µg/mL)	56.37 ± 0.45	>2500

**Table 2:** The contents of chemical markers, biological activities, Brix and acidity of MP1 and the placebo.

Biological Activities	MP1				Placebo
	1 Day	1 Month	2 Months	3 Months	1 Day
Total phenolic compounds (mg/L GAE)	184.00 ± 1.91	171.08 ± 2.92	173.17 ± 1.67	169.42 ± 0.42	3.78 ± 2.22
Anthocyanin content (mg/L CGE)	25.66 ± 0.32	22.33 ± 0.24	20.30 ± 0.05	19.33 ± 0.07	0.03 ± 0.00
FRAP activity (μM L-ascorbic acid equivalent)	602.40 ± 2.33	577.62 ± 1.95	574.55 ± 1.39	551.53 ± 1.10	5.49 ± 3.33
DPPH radical activity (IC_50_ µg/mL)	56.37 ± 0.45	75.44 ± 0.04	72.33 ± 0.21	71.80 ± 0.46	>2500
pH	3.36	3.37	3.37	3.40	3.37
Degree Brix	1.4	1.4	1.4	1.4	1.4

**Table 3:** The demographic data of the participants (N = 30). Data are presented as mean ± SD.

Demographic Data	MP1			Placebo			F-Test, *p*-Value
Age (years)	51.94	±	2.44	51.13	±	2.96	F(1.31) = 0.751, *p* = 0.393
Menstrual cessation (years)	2.00	±	1.58	1.25	±	1.39	F(1.31) = 2.083, *p* = 0.159
Weight (kg)	59.33	±	8.52	60.07	±	6.13	F(1.31) = 0.081, *p* = 0.778
BMI (kg/m^2^)	24.54	±	2.97	24.25	±	2.87	F(1.31) = 0.086, *p* = 0.772
Systolic BP (mmHg)	118.00	±	12.66	117.19	±	10.81	F(1.31) = 0.039, *p* = 0.845
Diastolic BP (mmHg)	74.71	±	8.54	74.13	±	6.97	F(1.31) = 0.045, *p* = 0.833
Heart rate (bpm)	71.88	±	11.44	70.38	±	10.41	F(1.31) = 0.156, *p* = 0.696
Education (years)	10.71	±	4.36	10.13	±	4.92	F(1.31) = 0.129, *p* = 0.722
No. of menopausal/perimenopausal women	13/3			9/7			*p* = 0.252

**Table 4:** The effect of MP1 on P300 and N100 at baseline, 1 month of treatment, 2 months of treatment and 1 month after cessation (N = 30/group). Data are presented as mean ± SD.

Location	ERP Wave	Baseline	1 Month		2 Months		1 Month After Cessation	
			MP1	Placebo	MP1	Placebo	MP1	Placebo
Fz	N100 Latency (msec)	96.77 ± 9.77	91.83 ± 12.91 * F(2.58) = 5.813 *p* = 0.004	102.20 ± 12.42	92.09 ± 12.78 F(2.58) = 1.293 *p* = 0.280	95.87 ± 13.00	93.07 ± 11.59 F(2.58) = 0.546, *p* = 0.463	95.60 ± 14.77
	N100 Amplitude (µV)	5.73 ± 2.28	6.65 ± 2.95 F(2.58) = 1.486, *p* = 0.283	6.82 ± 2.61	6.44 ± 2.64 F(2.58) = 1.346, *p* = 0.266	5.47 ± 2.09	7.90 ± 3.11 F(2.58) = 0.015, *p* = 0.905	8.00 ± 3.15
	P300 Latency (msec)	307.33 ± 10.63	295.50 * ± 8.12 F(2.58) = 14.065, *p* < 0.001	303.77 ± 7.55	296.57 ± 8.11 * F(2.58) = 11.739, *p* < 0.001	304.41 ± 7.68	298.57 ± 7.59 F(2.58) = 1.305, *p* = 0.258	300.87 ± 8.00
	P300 Amplitude (µV)	6.34 ± 1.88	6.89 ± 0.3.49 F(2.58) = 0.562, *p* = 0.572	6.22 ± 2.19	6.41 ± 1.80 F(2.58) = 0.035, *p* = 0.965	6.28 ± 1.89	6.06 ± 2.55 F(2.58) = 0.017, *p* = 0.897	5.97 ± 3.09
Cz	N100 Latency (msec)	98.63 ± 9.80	93.50 ± 13.76 F(2.58) = 2.432, *p* = 0.094	100.23 ± 13.13	90.76 ± 12.89 * F(2.58) = 4.059, *p* = 0.021	97.83 ± 12.40	95.17 ± 12.37 F(2.58) = 0.000, *p* = 0.985	95.23 ± 14.12
	N100 Amplitude (µV)	4.02 ± 2.07	5.15 ± 2.61 F(2.58) = 1.912, *p* = 0.172	6.15 ± 2.97	5.91 ± 2.54 F(2.58) = 0.487, *p* = 0.488	5.47 ± 2.37	6.00 ± 2.72 F(2.58) = 0.858, *p* = 0.358	6.64 ± 2.67
	P300 Latency (msec)	304.54 ± 11.47	297.10 ± 9.30 F(2.58) = 6.091, *p* = 0.03	305.03 ± 8.60 *	296.71 ± 8.22 F(2.58) = 8.314, *p* < 0.001	305.84 ± 8.07 *	299.73 ± 8.86 F(2.58) = 0.770, *p* = 0.384	301.63 ± 7.88
	P300 Amplitude (µV)	6.84 ± 2.65	6.68 ± 3.13 F(2.58) = 1.452, *p* = 0.240	5.71 ± 2.55	5.56 ± 2.54 F(2.58) = 0.000, *p* = 0.984	5.57 ± 2.04	6.53 ± 3.86 F(2.58) = 2.757, *p* = 0.762	6.24 ± 3.51

* *p*-value < 0.05, compared to placebo.

**Table 5:** The effect of MP1 on working memory at baseline, after 1 month and 2 months treatment and 1 month after cessation (N = 30/group). Data are presented as mean ± SD.

Cognitive Domain Test	Baseline	1 Month		2 Months		1 Month After Cessation	
		MP1	Placebo	MP1	Placebo	MP1	Placebo
Simple reaction time (ms)	657.38 ± 266.50	585.25 ± 115.91 F(2.58) = 2.212, *p* = 0.116	607.37 ± 127.86	566.51 ± 79.85 F(2.58) = 0.800, *p* = 0.375	586.10 ± 89.56	565.44 ± 72.55 F(2.58) = 0.773, *p* = 0.373	584.70 ± 95.47
Digit vigilance (ms)	657.27 ± 32.19	660.71 ± 48.65 F(2.58) = 0.073, *p* = 0.930	655.71 ± 45.12	659.43 ± 45.04 F(2.58) = 0.839, *p* = 0.364	648.64 ± 46.30	643.75 ± 50.21 F(2.58) = 0.723, *p* = 0.399	665.03 ± 127.53
Choice reaction time (ms)	866.80 ± 196.10	784.47 ± 87.31 F(2.58) = 0.546, *p* = 0.455	808.15 ± 125.01	773.95 ± 99.25 F(2.58) = 0.364, *p* = 0.549	759.81 ± 81.39	738.18 ± 155.38 F(2.58) = 0.020, *p* = 0.889	743.74 ± 152.06
Accuracy of digit vigilance (%)	64.22 ± 22.08	75.79 ± 22.12 F(2.58) = 0.000, *p* = 0.987	75.88 ± 22.25	80.64 ± 21.05 F(2.58) = 0.988, *p* = 0.324	85.55 ± 16.71	80.43 ± 25.91 F(2.58) = 0.316, *p* = 0.576	83.59 ± 16.58
Accuracy of choice reaction (%)	96.80 ± 2.66	97.93 ± 2.45 F(2.58) = 4.860, *p* = 0.032	96.40 ± 3.20	97.60 ± 2.70 F(2.58) = 1.812, *p* = 0.183	96.47 ± 3.74	97.53 ± 2.45 F(2.58) = 0.788, *p* = 0.378	96.87 ± 3.31
Delayed word recognition (ms)	1459.70 ± 298.52	1161.12 ± 200.46 F(2.58) = 0.240, *p* = 0.626	1135.41 ± 199.03	1136.02 ± 187.10 F(2.58) = 0.118, *p* = 0.733	1119.08 ± 189.02	1135.27 ± 190.38 F(2.58) = 0.000, *p* = 0.997	1135.45 ± 205.71
Delayed picture recognition (ms)	1443.97 ± 178.40	1229.53 ± 202.46 F(2.58) = 0.033, *p* = 0.856	1220.16 ± 196.08	1204.93 ± 235.23 F(2.58) = 0.247, *p* = 0.588	1239.09 ± 250.01	1194.47 ± 222.05 F(2.58) = 0.678, *p* = 0.410	1243.94 ± 239.85
Spatial memory (ms)	1604.74 ± 394.94	1233.00 ± 215.48 F(2.58) = 5.219, *p* = 0.026	1389.79 ± 324.47	1220.65 ± 217.69 F(2.58) = 0.118, *p* = 0.732	1239.91 ± 215.22	1240.04 ± 230.93 F(2.58) = 0.013, *p* = 0.911	1233.24 ± 237.62
Numeric working memory (ms)	1209.06 ± 216.96	1050.78 ± 202.65 F(2.58) = 0.055, *p* = 0.815	1040.24 ± 139.67	1034.47 ± 161.00 F(2.58) = 1.274, *p* = 0.264	989.66 ± 146.13	999.33 ± 157.55 F(2.58) = 0.595, *p* = 0.444	1032.13 ± 171.59
Accuracy of word recognition (%)	83.79 ± 9.05	84.14 ± 7.95 F(2.58) = 0.883, *p* = 0.351	86.09 ± 7.87	83.10 ± 10.04 F(2.58) = 0.002, *p* = 0.963	83.22 ± 9.29	82.53 ± 8.48 F(2.58) = 1.103, *p* = 0.298	84.83 ± 8.19
Accuracy of picture recognition (%)	81.22 ± 10.68	82.33 ± 12.02 F(2.58) = 0.163, *p* = 0.668	80.77 ± 10.95	88.67 ± 12.03 F(2.58) = 0.081, *p* = 0.077	87.83 ± 10.64	89.17 ± 13.14 F(2.58) = 0.120, *p* = 0.724	90.34 ± 12.10
Accuracy of spatial memory (%)	78.98 ± 14.64	89.26 ± 10.49 F(2.58) = 0.438, *p* = 0.235	85.10 ± 15.88	87.60 ± 13.47 F(2.58) = 0.211, *p* = 0.647	89.35 ± 15.95	87.68 ± 11.71 F(2.58) = 012, *p* = 0.912	87.30 ± 14.68
Accuracy of numeric working memory (%)	89.89 ± 9.19	89.01 ± 14.67 F(2.58) = 0.190, *p* = 0.665	90.45 ± 10.53	91.78 ± 11.99 F(2.58) = 0.635, *p* = 0.851	91.22 ± 10.88	90.45 ± 12.06 F(2.58) = 1.069, *p* = 0.306	91.88 ± 9.12

*p*-value < 0.05, compared to placebo.

**Table 6:** The effect of “MP1” on blood chemistry profiles (N = 30). Data are presented as mean ± SD. *, *p*-value < 0.05, compared with the placebo group.

Blood Chemistry	References	Baseline	1 Month		2 Months	
			MP1	Placebo	MP1	Placebo
Glucose	70–100 mg/dL	84.3 ± 8.78	81.19 ± 6.09 F(2.58) = 2.121, *p* = 0.156	84.25 ± 5.80	86.25 ± 8.28 F(2.58) = 0.733, *p* = 0.399	84.00 ± 6.47
BUN	5.8–19.1 mg/dL	16.29 ± 18.02	11.35 ± 3.10 F(2.58) = 0.293, *p* = 0.593	10.81 ± 2.48	11.04 ± 2.94 F(2.58) = 0.032, *p* = 0.860	10.86 ± 2.60
Creatinine	0.5–1.5 mg/dL	0.74 ± 0.13	0.71 ± 0.13 F(2.58) = 230, *p* = 0.635	0.69 ± 0.08	0.76 ± 0.17 F(2.58) = 2.924, *p* = 0.098	0.68 ± 0.09
Sodium	130–147 mEq/L	140.38 ± 2.39	141.13 ± 2.06 F(2.58) = 2.897, *p* = 0.099	139.69 ± 2.68	139.25 ± 2.27 F(2.58) = 0.000, *p* = 1.000	139.25 ± 2.11
Potassium	3.4–4.7 mEq/L	4.54 ± 0.41	4.42 ± 0.45 F(2.58) = 0.111, *p* = 0.741	4.46 ± 0.27	4.52 ± 0.32 F(2.58) = 1.528, *p* = 0.226	4.39 ± 0.09
Bicarbonate	20.6–28.3 mEq/L	25.29 ± 2.56	24.87 ± 2.35 F(2.58) = 9.358, *p* = 0.005	27.03 ± 1.55 *	26.16 ± 1.54 F(2.58) = 0.264, *p* = 0.611	25.82 ± 2.13
Chloride	96–107 mEq/L	100.19 ± 2.26	101.19 ± 2.29 F(2.58) = 0.089, *p* = 0.768	100.94 ± 2.46	100.69 ± 2.44 F(2.58) = 0.111, *p* = 0.742	100.44 ± 1.75
Albumin	3.8–5.4 g/dL	4.39 ± 4.36	4.36 ± 0.25 F(2.58) = 0.305, *p* = 0.585	4.41 ± 0.19	4.37 ± 0.21 F(2.58) = 0.044, *p* = 0.836	4.39 ± 0.17
Total bilirubin	0.3–1.5 mg/dL	0.64 ± 0.38	0.59 ± 0.25 F(2.58) = 1.606, *p* = 0.215	0.69 ± 0.23	0.64 ± 0.38 F(2.58) = 0.605, *p* = 0.443	0.56 ± 0.17
Direct bilirubin	0.0–0.5 mg/dL	0.21 ± 0.09	0.19 ± 0.07 F(2.58) = 0.350, *p* = 0.559	0.18 ± 0.04	0.21 ± 0.08 F(2.58) = 0.550, *p* = 0.465	0.19 ± 0.06
ALT	4–36 U/L	15.56 ± 7.47	13.88 ± 4.56 F(2.58) = 2.462, *p* = 0.127	16.50 ± 4.90	14.31 ± 5.53 F(2.58) = 0.537, *p* = 0.469	15.8 ± 4.96
AST	12–32 U/L	21.75 ± 4.99	19.88 ± 3.20 F(2.58) = 0.801, *p* = 0.378	18.81 ± 3.51	21.31 ± 5.15 F(2.58) = 0.397, *p* = 0.534	20.19 ± 4.96
LDH	89–221 U/L	212.44 ± 41.73	204.19 ± 43.89 F(2.58) = 1.235, *p* = 0.275	187.13 ± 42.94	214.25 ± 39.27 F(2.58) = 0.860, *p* = 0.361	199.63 ± 49.39
CK-MB	0–25 U/L	15.88 ± 7.47	17.31 ± 4.41 F(2.58) = 0.298, *p* = 0.589	16.50 ± 4.00	18.13 ± 5.76 F(2.58) = 0.032, *p* = 0.860	17.80 ± 4.44
Cholesterol	127–262 mg/dL	223.38 ± 44.6	217.19 ± 55.26 F(2.58) = 0.259, *p* = 0.615	207.94 ± 47.28	204.94 ± 29.85 F(2.58) = 0.009, *p* = 0.925	203.69 ± 43.01
Triglyceride	10–200 mg/dL	122.80 ± 73.86	96.06 ± 34.55 F(2.58) = 0.217, *p* = 0.645	101.88 ± 36.08	109.25 ± 58.22 F(2.58) = 0.118, *p* = 0.0.734	115.63 ± 46.20
HDL-C	>35 mg/dL	66.38 ± 29.26	68.81 ± 27.59 F(2.58) = 878, *p* = 0.356	61.88 ± 10.78	66.69 ± 27.08 F(2.58) = 1.229, *p* = 0.276	58.44 ± 12.35
LDL-CHOL (DIRECT)	10–150 mg/dL	148.88 ± 40.92	146.63 ± 45.53 F(2.58) = 0.175, *p* = 0.679	153.19 ± 43.17	142.00 ± 34.17 F(2.58) = 0.166, *p* = 0.687	147.44 ± 12.35

Reference value: Laboratory unit, Srinagarind Hospital, Faculty of Medicine, Khon Kaen University. AST = Aspartate Aminotransferase; ALT = Alanine Aminotransferase; LDH = Lactate Dehydrogenase; CK-MB = Creatine kinase-MB, HDL-C = high-density lipoprotein cholesterol; LDL-CHOL = low-density lipoprotein cholesterol.

**Table 7:** The effect of MP1 on hematological changes (N-30). Data are presented as mean ± SD.

Hematology	References	Baseline	1 Month		2 Months	
			MP1	Placebo	MP1	Placebo
Hb	13.0–16.7 g/dL	12.81 ± 0.26	12.64 ± 0.24 F(2.58) = 1.356, *p* = 0.253	12.2 ± 0.30	12.72 ± 0.28 F(2.58) = 2.891, *p* = 0.099	12.03 ± 0.30
HCT	40.5–50.8%	38.87 ± 0.76	38.43 ± 0.61 F(2.58) = 1.365, *p* = 0.252	37.0 ± 1.00	38.63 ± 0.82 F(2.58) = 1.755, *p* = 0.195	37.06 ± 0.85
WBC	4.6–10.6 10^3^/μL	5.84 ± 0.23	5.50 ± 0.31 F(2.58) = 1.890, *p* = 0.179	6.2 ± 0.50	5.88 ± 0.37 F(2.58) = 0.006, *p* = 0.938	5.85 ± 0.31
PLT	173–383 10^3^/μL	265 ± 10.56	252.52 ± 13.93 F(2.58) = 1.394, *p* = 0.244	276.65 ± 14.82	255.65 ± 10.25 F(2.58) = 1.117, *p* = 0.296	273.87 ± 12.37
RBC	4.7–6.2 10^6^/μL	4.59 ± 0.10	4.52 ± 0.11 F(2.58) = 0.177, *p* = 0.677	4.5 ± 0.10	4.58 ± 0.08 F(2.58) = 1.091, *p* = 0.304	4.44 ± 0.10
MCV	80.0–97.8 fL	85.11 ± 2.08	85.59 ± 1.88 F(2.58) = 0.460, *p* = 0.503	83.4 ± 2.62	84.64 ± 1.96 F(2.58) = 0.049, *p* = 0.827	83.98 ± 2.31
MCH	25.2–32.0 pg	28.05 ± 0.69	28.19 ± 0.72 F(2.58) = 0.515, *p* = 0.479	27.4 ± 0.85	27.84 ± 0.62 F(2.58) = 0.286, *p* = 0.596	27.28 ± 0.86
MCHC	31.3–33.4 g/dL	32.97 ± 0.28	32.89 ± 0.18 F(2.58) = 0.006, *p* = 0.940	32.9 ± 0.17	32.93 ± 0.24 F(2.58) = 2.837, *p* = 0.103	32.43 ± 0.18
RDW	11.9–14.8%	14.98 ± 0.48	14.42 ± 0.29 F(2.58) = 0.422, *p* = 0.521	14.9 ± 0.66	14.40 ± 0.42 F(2.58) = 0.264, *p* = 0.611	14.84 ± 0.74
NE%	43.7–70.9%	48.83 ± 1.70	48.51 ± 1.73 F(2.58) = 0.058, *p* = 0.811	47.8 ± 2.20	49.84 ± 1.88 F(2.58) = 0.022, *p* = 0.883	49.50 ± 1.28
LY%	20.1–44.5%	41.14 ± 1.62	41.24 ± 1.99 F(2.58) = 001, *p* = 0.976	41.2 ± 1.75	40.78 ± 2.18 F(2.58) = 0.453, *p* = 0.506	39.18 ± 0.96
MO%	3.4–9.8%	6.09 ± 0.53	5.89 ± 0.63 F(2.58) = 0.181, *p* = 0.674	5.6 ± 0.36	6.11 ± 0.53 F(2.58) = 0.500, *p* = 0.485	6.55 ± 0.34
EO%	0.7–9.2%	3.34 ± 0.68	3.45 ± 0.89 F(2.58) = 0.713, *p* = 0.405	4.9 ± 1.42	2.72 ± 0.52 F(2.58) = 1.583, *p* = 0.218	4.18 ± 1.04
BA%	0.0–2.6%	0.60 ± 0.13	0.48 ± 0.06 F(2.58) = 0.666, *p* = 0.421	0.6 ± 0.06	0.56 ± 0.09 F(2.58) = 0.059, *p* = 0.811	0.58 ± 0.06

Reference value: Laboratory unit, Srinagarind Hospital, Faculty of Medicine, Khon Kaen University. Hb (hemoglobin), HCT (hematocrit), WBC (white blood cell), PLT (platelet), RBC (red blood cell), MCV (mean corpuscular volume), MCH (mean corpuscular hemoglobin), MCHC (mean corpuscular hemoglobin concentration), RDW (red cell distribution width), NE (neutrophil), LY (lymphocyte), MO (monocyte), EO (eosinophil), BA (basophil).

**Table 8:** The adverse effects that subjects presented with during the interventions.

	1 Month		2 Months	
Event	MP1	Placebo	MP1	Placebo
Breast tenderness	2(6.67%)	1(3.33%)	2(6.67%)	1(3.33%)
Severity	mild (1), moderate (1)	moderate (1)	mild (2)	moderate (1)
Hot flash	3(10%)	8(26.67%)	1(3.33%)	2(6.67%)
Severity	moderate (3)	mild (5), moderate (3)	mild (1)	mild (1), moderate (1)
Perspiration	2(6.67%)	1(3.33%)	1(3.33%)	0
Severity	moderate (2)	moderate (1)	mild (1)	-
Mood swings	1(3.33%)	0	0	0
Severity	moderate (1)	-	-	-
Dizziness	1(3.33%)	1(3.33%)	0	0
Severity	mild (1)	mild (1)	-	-

## Data Availability

The data presented in this study are available on request from the corresponding author.

## Supplementary Materials

The following supporting information can be downloaded at: https://www.mdpi.com/article/10.3390/antiox14101262/s1. Supplementary File S1: Physical activity measurement; Supplementary File S2: Food consumption assessment; a blank written consent form is provided in Supplementary Material File S3.
