# Supplementary material for: A Randomized Controlled Trial on the Safety and Cognitive Benefits of a Novel Functional Drink from a Purple Waxy Corn Byproduct in Peri- and Postmenopausal Women
Source: Antioxidants (Basel). 2025 Oct 20;14(10):1262. doi: 10.3390/antiox14101262 (PMC12561858; doi:10.3390/antiox14101262)
Supplement: Supplementary file 1 [file antioxidants-14-01262-s001.zip › antioxidants-3879342-supplementary/S1 Physical Activity Questionnaire.pdf]

Physical Activity Questionnaire

Medical Record Number: _______

Patient Code: _______Date: _______

Visit: _______ ID Number: _______

Questionnaire for Physical Activity Assessment in Elderly Patients (Physical Activity Questionnaire)

Instructions to Respondent: This questionnaire is designed to collect information about your physical activity patterns to assess your current physical activity levels and related behaviors. The questions focus on activities you may have performed during the past week. Please answer all questions honestly based on your actual experiences over the past seven days.

Please mark ✓ in the designated field ☐ that corresponds to the practices you have followed during the past week.

1. During the past week, how often did you engage in vigorous physical activities that significantly increased your heart rate during leisure time? (vigorous activities such as running, vigorous cycling, vigorous swimming, activities lasting more than 10 minutes that made you breathe much harder than normal, such as carrying heavy loads, fast walking, climbing stairs)?

☐ Did not engage ☐ 1 time/day ☐ 2 time/day ☐ 3 time/day ☐ More than 3 time/day (please specify number of times: _______)

☐ 1-2 days per week ☐ 3-4 days per week ☐ 5-6 days per week ☐ More than 6 days per week

1.1 On days when you engaged in vigorous physical activities, how much time did you spend?

☐ 10 minutes ☐ 10-20 minutes ☐ 20-30 minutes ☐ 30-40 minutes ☐ 40-50 minutes ☐ 1 hour ☐ 50-60 minutes ☐ 1 hour or more

2 During the past week, how often did you engage in moderate physical activities during leisure time (moderate activities such as cycling at regular pace lasting more than 10

minutes that made you breathe somewhat harder than normal, such as carrying light loads)?

☐ Did not engage ☐ 1 time/day ☐ 2 time/day ☐ 3 time/day ☐ More than 3 time/day (please specify number of times: _______)

☐ 1-2 days per week ☐ 3-4 days per week ☐ 5-6 days per week ☐ More than 6 days per week

2.1 On days when you engaged in moderate physical activities, how much time did you spend?

☐ 10 minutes ☐ 10-20 minutes ☐ 20-30 minutes ☐ 30-40 minutes ☐ 40-50 minutes ☐ 1 hour ☐ 50-60 minutes ☐ 1 hour or more

3. During the past week, how often did you engage in walks for leisure during your free time (walking at a comfortable pace, walking for exercise, walking for relaxation, walking to exercise pets, walking around markets)?

☐ Did not engage ☐ 1 time/day ☐ 2 time/day ☐ 3 time/day ☐ More than 3 time/day (please specify number of times: _______)

☐ 1-2 days per week ☐ 3-4 days per week ☐ 5-6 days per week ☐ More than 6 days per week

3.1 On days when you engaged in moderate physical activities, how much time did you spend?

☐ 10 minutes ☐ 10-20 minutes ☐ 20-30 minutes ☐ 30-40 minutes ☐ 40-50 minutes ☐ 1 hour ☐ 50-60 minutes ☐ 1 hour or more

4. During the past week, how often did you engage in sitting (sitting at work, sitting while relaxing, sitting while reading books, sitting while watching television)?

☐ Did not engage ☐ 1 time/day ☐ 2 time/day ☐ 3 time/day ☐ More than 3 time/day (please specify number of times: _______)

☐ 1-2 days per week ☐ 3-4 days per week ☐ 5-6 days per week ☐ More than 6 days per week

4.1 On days when you engaged in sitting, how much time did you spend?

☐ 10 minutes ☐ 10-20 minutes ☐ 20-30 minutes ☐ 30-40 minutes ☐ 40-50 minutes ☐ 1 hour ☐ 50-60 minutes ☐ 1 hour or more
