# Supplementary material for: A Randomized Controlled Trial on the Safety and Cognitive Benefits of a Novel Functional Drink from a Purple Waxy Corn Byproduct in Peri- and Postmenopausal Women
Source: Antioxidants (Basel). 2025 Oct 20;14(10):1262. doi: 10.3390/antiox14101262 (PMC12561858; doi:10.3390/antiox14101262)
Supplement: Supplementary file 1 [file antioxidants-14-01262-s001.zip › antioxidants-3879342-supplementary/S2 Dietary questionaire and Result.pdf]

Dietary Questionnaire

Patient Information:

Medical record number: ____________Patient name: ____________

Date: ____________Visit: ____________ ID number: ____________

This questionnaire aims to collect information about your eating habits during the past week to help assess your nutritional intake and eating patterns. Please answer all questions honestly. If you cannot remember exactly, please provide your best estimate based on your usual eating habits during the past week.

Basic Information About Your Current Eating Habits:

Please mark ✓ in the designated field ☐ that corresponds to the practices you have followed during the past week.

1. During the past week, how often did you eat rice?

   ☐ Did not eat ☐ 1 time per day ☐ 2 times per day ☐ 3 times per day

   ☐ More than 3 times per day

   ☐ 1-2 times per week ☐ 3-4 times per week ☐ 4-5 times per week ☐ 6-7 times per week

   1.1 Please specify the amount consumed

☐ Less than half a plate ☐ Half a plate to one plate ☐ One plate to one and a half plates

☐ More than one and a half plates

2. During the past week, how often did you consume meat?

   ☐ Did not eat ☐ 1 time per day ☐ 2 times per day ☐ 3 times per day

   ☐ More than 3 times per day

   ☐ 1-2 times per week ☐ 3-4 times per week ☐ 4-5 times per week ☐ 6-7 times per week

   2.1 Please specify the amount consumed

☐ Less than half a plate ☐ Half a plate to one plate ☐ One plate to one and a half plates

☐ More than one and a half plates

3. During the past week, how often did you consume egg?

☐ Did not eat ☐ 1 time per day ☐ 2 times per day ☐ 3 times per day

☐ More than 3 times per day

☐ 1-2 times per week ☐ 3-4 times per week ☐ 4-5 times per week ☐ 6-7 times per week

3.1 Please specify the amount consumed

☐ Less than half an egg ☐ Half an egg to an egg ☐ 2 eggs ☐ 3 eggs ☐ More than 3 eggs

4. During the past week, how often did you drink milk?

☐ Did not eat ☐ 1 time per day ☐ 2 times per day ☐ 3 times per day

☐ More than 3 times per day

☐ 1-2 times per week ☐ 3-4 times per week ☐ 4-5 times per week ☐ 6-7 times per week

4.1 Please specify the amount consumed

☐ Less than half of glass ☐ Half a glass to one glass ☐ 2 glasses ☐ 3 glasses

☐ More than 3 glasses

5. During the past week, how often did you consume coconut curry?

☐ Did not eat ☐ 1 time per day ☐ 2 times per day ☐ 3 times per day

☐ More than 3 times per day

☐ 1-2 times per week ☐ 3-4 times per week ☐ 4-5 times per week

☐ 6-7 times per week

5.1 Please specify the amount consumed

☐ Less than half cup ☐ Half cup to a cup ☐ 1 cup to 1½ cups

☐ More than 1½ cups

6. During the past week, how often did you consume vegetables?

☐ Did not eat meals ☐ 1 time per day ☐ 2 times per day ☐ 3 times per day

☐ More than 3 times per day

☐ 1-2 times per week ☐ 3-4 times per week ☐ 4-5 times per week

☐ 6-7 times per week

6.1 Please specify the amount consumed

☐ Less than half a plate ☐ Half a plate to one plate ☐ One plate to one and a half plates

7. During the past week, how often did you consume fruits?

☐ Did not eat meals ☐ 1 time per day ☐ 2 times per day ☐ 3 times per day

☐ More than 3 times per day

☐ 1-2 times per week ☐ 3-4 times per week ☐ 4-5 times per week

☐ 6-7 times per week

7.1 Please specify the amount consumed

☐ Less than half a plate ☐ Half a plate to one plate ☐ One plate to one and a half plates

8. During the past week, how often did you consume desserts?

☐ Did not eat meals ☐ 1 time per day ☐ 2 times per day ☐ 3 times per day

☐ More than 3 times per day

☐ 1-2 times per week ☐ 3-4 times per week ☐ 4-5 times per week

☐ 6-7 times per week

8.1 Please specify the amount consumed

☐ Less than half cup ☐ 1 cup ☐ 2 cup ☐ More than 2 cups

9. During the past week, how often did you consume snacks?

☐ Did not eat meals ☐ 1 time per day ☐ 2 times per day ☐ 3 times per day

☐ More than 3 times per day

☐ 1-2 times per week ☐ 3-4 times per week ☐ 4-5 times per week

☐ 6-7 times per week

9.1 Please specify the amount consumed

☐ Less than half cup ☐ 1 plate ☐ 2 plate ☐ More than 2 plate

Dietary consumption during intervention and 1-month after the intervention cessation period

Figure S1 showed dietary consumption at 1, 2-month intervention period and 1-month cessation period. The current data demonstrated that no significant changes of this parameters among groups throughout the study period.

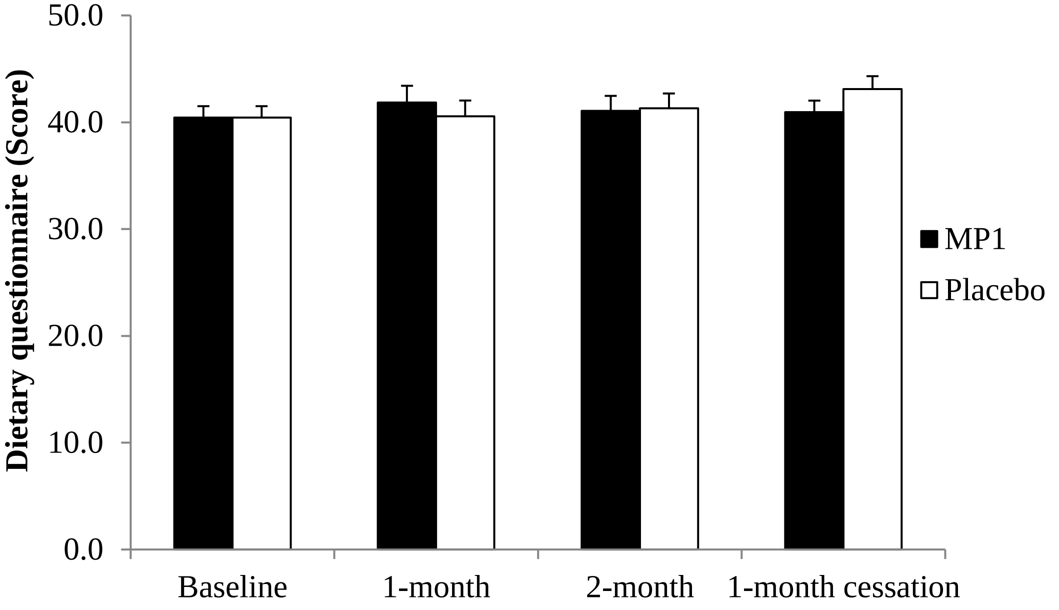

Figure S1 The dietary score of participant who consumed MP1 during the intervention period and 1-month cessation period (N=30/group).
