# Supplementary material for: A Randomized Controlled Trial on the Safety and Cognitive Benefits of a Novel Functional Drink from a Purple Waxy Corn Byproduct in Peri- and Postmenopausal Women
Source: Antioxidants (Basel). 2025 Oct 20;14(10):1262. doi: 10.3390/antiox14101262 (PMC12561858; doi:10.3390/antiox14101262)
Supplement: Supplementary file 1 [file antioxidants-14-01262-s001.zip › antioxidants-3879342-supplementary/S3 VOLUNTEER INFORMATION SHEET and Consent form.pdf]

VOLUNTEER INFORMATION SHEET

Research Project Title (Thai): Development and Evaluation of Cognitive Enhancement Product (MP1) made from Selected Thai Herbs for Postmenopausal Women

Principal Investigator: Miss Woranan Kirisattayakul

Co-Principal Investigator: .....

INTRODUCTION

Purple corn cob and pandan leaves are plants that can supplement yin energy according to Traditional Chinese Medicine principles or are plants with cooling properties suitable for postmenopausal women who are found to have yin energy deficiency in their bodies. This condition causes many abnormalities including memory impairment in this population group. Therefore, the researchers have developed a memory enhancement product from Thai herbs for postmenopausal women in the form of a beverage containing purple corn cob extract and pandan leaves as main components (MP1 memory enhancement health drink product). The product was found to inhibit memory impairment in experimental rats simulating menopause by ovariectomy, as well as being able to inhibit free radicals, enhance the function of antioxidants in the body, and inhibit the activity of enzymes that destroy neurotransmitters related to learning and memory in the brain. From the aforementioned data, it can be seen that MP1 product may be a health supplement that can delay and inhibit memory impairment in postmenopausal women consumers. However, there are currently no scientific reports regarding this issue. Therefore, the researchers have conducted this research project to study the memory enhancement effects of MP1 memory enhancement health drink product in postmenopausal women volunteers, including emotional and psychological changes, and changes in free radical balance.

RESEARCH OBJECTIVES

Primary Objective:

- To study the effects of MP1 memory enhancement health drink product in delaying memory impairment in postmenopausal women volunteers, evaluated by measuring brain waves through auditory stimulation

Secondary Objectives:

- To study the effects of MP1 memory enhancement health drink product on memory changes tested by computer memory measurement programs and changes in brain blood flow
- To study the effects of MP1 memory enhancement health drink product on emotional and psychological changes in postmenopausal women volunteers
- To study the effects of MP1 memory enhancement health drink product on changes in free radical levels and antioxidants, the function of acetylcholinesterase enzyme, monoamine oxidase enzyme types A and B, and cortisol hormone levels in postmenopausal women volunteers
- To evaluate the safety of MP1 memory enhancement health drink in postmenopausal women volunteers
- To study side effects that may be related to MP1 memory enhancement health drink product in postmenopausal women volunteers

Your participation in this research project is voluntary. If you do not wish to participate, there will be no adverse effects, and if you decide to participate in this study, you can withdraw from the project at any time without any adverse effects.

PROCEDURES IF YOU PARTICIPATE IN THE RESEARCH PROJECT

If you decide to participate in the research and provide your signature as evidence on the volunteer consent form, you will undergo volunteer screening procedures. You will receive a physical examination conducted by a medical practitioner who serves as a co-investigator, and blood samples will be collected for blood chemistry measurements including liver function values, kidney function values, cholesterol and triglyceride levels in blood, hematological values, and blood electrolyte values. Blood will be collected from a vein at the elbow bend area with a volume of 10 milliliters (approximately 2/3 tablespoon). Subsequently, intelligence level assessment will be conducted using intelligence evaluation forms, brain wave measurements will be taken through auditory stimulation, and memory will be assessed using computer programs. Following these assessments, electrocardiogram measurements will be performed along with blood pressure and respiratory rate monitoring. Brain blood circulation will be measured, and mental health changes will be evaluated using two sets of questionnaires. The entire process from blood collection through completion of all tests will require approximately 2 hours.

Upon completion of the screening process, you will be randomly assigned to groups using a computer system to determine which type of beverage you will receive first. There will be two types: MP1 or a beverage that has the same color, smell, and taste as MP1 beverage. You will be required to drink each type of beverage continuously for 12 weeks with an 8-week interval before switching to drinking the remaining beverage type for another 12 weeks. Details regarding measurements and the number of times researchers will schedule appointments for your evaluation will be presented in the diagram and table on page 3.

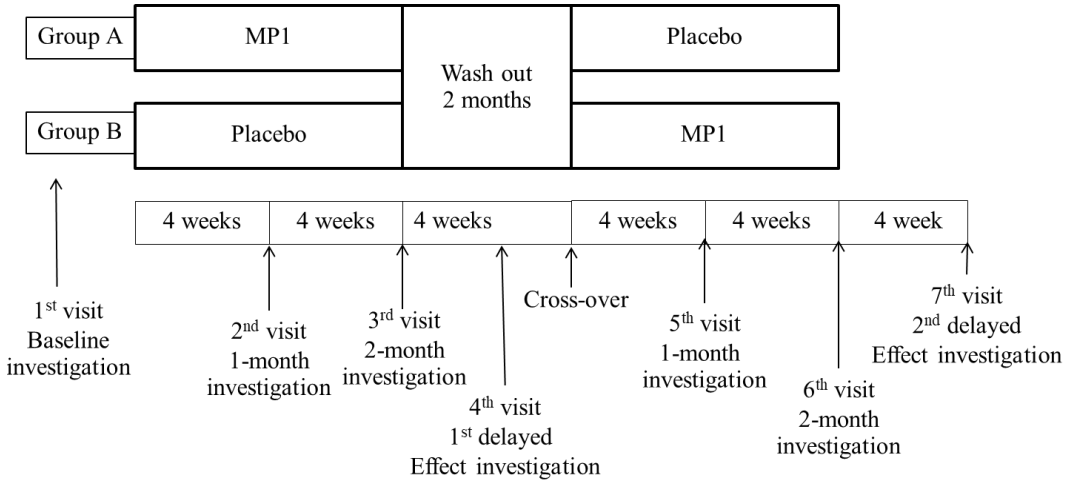

Assessment Schedule, Procedures and Visit Frequency

Assessment Procedure	Assessment Method	Visit Frequency
Intelligence assessment using intelligence evaluation questionnaire	Computer-based intelligence assessment questionnaire	1 time (screening only) Taking approximately 15 minutes
Memory assessment by computer program	Computer memory assessment using attention and memory tasks. Volunteers respond to stimuli appearing on screen by pressing appropriate keys	7 times (screening and all assessment visits) Taking 30 minutes per assessment
Computer-based memory testing	Computer-based memory assessment with stimulus presentation. Volunteers respond with Yes/No button presses. Computer records accuracy and response time for various memory tasks	7 times (screening and all assessment visits) Taking 15 minutes per assessment
Brain blood flow measurement	Measurement using small ultrasound probe placed at temporal region to assess cerebral arterial blood flow velocity providing real-time blood flow information in brain arteries. Safe and painless	3 times (screening, visit 2 and visit 5) Taking 15 minutes per assessment
Mental health assessment	Two sets of questionnaires	7 times (screening and all assessment visits) Taking 15 minutes per assessment
Memory assessment using computer program, measurements and physiological monitoring	Computer-based memory assessment with physiological monitoring including heart rate variability, blood pressure, and other vital signs during cognitive tasks	7 times (screening and all assessment visits) Taking 10 minutes per assessment

POTENTIAL RISKS AND/OR DISCOMFORTS

The potential impacts that may occur to research participants in this study include allergic reactions and side effects that may arise from the phytoestrogenic activity of the product. However, you will receive care throughout the project through verbal telephone interviews and assessment forms to evaluate understanding of the research work and inquire about symptoms at that time. If any abnormalities occur, you will contact physicians including gynecologists, radiologists, and psychiatrists who are co-researchers in the project to provide care and treatment for such impacts, and they will bear all costs for treatment and medical care, including compensation for other damages if any. Additionally, since this experiment involves blood collection for various blood tests, there may be risks or discomforts from blood collection such as bleeding at the puncture site, fainting during blood collection, and blood clots at the puncture site. However, the researchers have arranged for professional nurses who are skilled and experienced in blood collection to perform the blood collection for you to reduce such risks or discomforts.

BENEFITS TO VOLUNTEERS

Direct benefits from participating in this research: If the MP1 beverage used in the research shows positive effects, it may help delay your memory impairment. Alternatively, you may not receive direct benefits from participating in this research, but the data obtained from the research will benefit the development of health-beneficial foods in the future. Additionally, you will receive physical examinations from medical practitioners.

RESEARCH COSTS/TRAVEL COMPENSATION/TIME COMPENSATION

You will receive travel expenses for participating in this research project at 100 baht per visit, totaling 700 baht that you will receive throughout the entire project.

CONFIDENTIALITY

During participation in this study, you are not required to answer questions you do not wish to answer. Data obtained from your answers and examinations will be combined with data from other volunteers participating in the study, and your data will be kept confidential. We will use codes instead of your name-surname in data recording and when searching for your name. In this study, only the research team will have access to the data. If we publish the study results in medical journals, we will not identify your name under any circumstances.

If you have questions, please ask us immediately. If you have problems or concerns later or want to know your test results from this research, you can contact Miss Woranan Kirisattayakul at telephone number (043) 363861 and mobile phone number 089-8332519, and Assoc. Prof. Dr. Jintanaporn Wattanathorn (advisor) at telephone number (043) 363540, mobile phone number 081-8721809, Department of Physiology, Faculty of Medicine, Khon Kaen University. If you have problems or concerns about your rights while participating in this study, please contact the Office of Human Research Ethics Committee, Khon Kaen University (Branch Office), Room 5317, 3rd Floor, Medical Education Building, Faculty of Medicine, Khon Kaen University, Phone: 089-7141913, Extension: 67133-4.

Note:

Researchers should provide copies of the volunteer consent form and volunteer information sheet, one set each, to volunteers or guardians.

When clinical research (whether for treatment or not) involves volunteers who require consent from legally authorized representatives (such as minors or patients with severe dementia), volunteers should receive appropriate explanations about the research that they can understand, and if possible, volunteers should sign and date the consent form themselves.

VOLUNTEER CONSENT FORM

I, (Mrs./Miss) ..... Surname ..... Age ..... years

Address ..... Village ..... Sub-district ..... District ..... Province .....

Identification Card Number ..... (Copy attached)

Regarding participation as a volunteer in the research project "Development and Evaluation of Memory Enhancement Product (MP1) from Thai Herbs for Postmenopausal Women," have received information from ..... (name of information provider) and have been informed about the research project details regarding:

- Research objectives and duration
- Procedures and methods that I must follow
- Benefits that I will receive
- Side effects or dangers that may arise from participating in the project

I understand that I can withdraw from this study at any time if I wish, without losing any rights to medical treatment that may occur subsequently, either currently or in the future, at this medical facility or other medical institutions. If any harm or damage occurs to me as a result of the research, the researchers and/or research sponsors will provide medical treatment and bear all costs for medical care, including compensation for other damages if any.

I have read and understood the above explanation and hereby consent to be a volunteer in this research project.

Volunteer Signature ..... (.....)

Information Provider Signature ..... (.....)

Witness ..... (not the explainer) (.....)

Date ..... Month ..... B.E. .....

Note: (1) Witnesses must not be physicians or researchers (2) Information providers/explainers must not be physician-researchers to prevent participation due to deference (3) In cases where volunteers cannot read/sign their name, use thumbprint as follows:

I cannot read, but the researcher has read the content of this consent form to me until I understand well. I therefore voluntarily place my right thumbprint on this consent form.

Information Provider Signature ..... (.....)

Witness ..... (not the explainer) (.....)

Date ..... Month ..... B.E. .....

Right Thumbprint
